# N-Acetylcysteine Treatment May Compensate Motor Impairments through Dopaminergic Transmission Modulation in a Striatal 6-Hydroxydopamine Parkinson’s Disease Rat Model

**DOI:** 10.3390/antiox12061257

**Published:** 2023-06-11

**Authors:** Rita Caridade-Silva, Bruna Araújo, Joana Martins-Macedo, Fábio G. Teixeira

**Affiliations:** 1 Life and Health Sciences Research Institute (ICVS), School of Medicine, University of Minho, 4710-057 Braga, Portugal; rcaridade@i3s.up.pt (R.C.-S.); bruna.araujo@i3s.up.pt (B.A.); 2 ICVS/3B’s—PT Government Associate Laboratory, 4710-057/4805-017 Braga/Guimarães, Portugal; 3 I3S—Instituto de Investigação e Inovação em Saúde, Universidade do Porto, 4200-135 Porto, Portugal; joanamace@gmail.com; 4 Center for Translational Health and Medical Biotechnology Research, School of Health, Polytechnic University of Porto, 4200-465 Porto, Portugal

**Keywords:** N-acetylcysteine, neuroprotection, disease modification

## Abstract

Preventing degeneration and the loss of dopaminergic neurons (DAn) in the brain while mitigating motor symptoms remains a challenge in Parkinson’s Disease (PD) treatment development. In light of this, developing or repositioning potential disease-modifying approaches is imperative to achieve meaningful translational gains in PD research. Under this concept, N-acetylcysteine (NAC) has revealed promising perspectives in preserving the dopaminergic system capability and modulating PD mechanisms. Although NAC has been shown to act as an antioxidant and (neuro)protector of the brain, it has yet to be acknowledged how this repurposed drug can improve motor symptomatology and provide disease-modifying properties in PD. Therefore, in the present work, we assessed the impact of NAC on motor and histological deficits in a striatal 6-hydroxydopamine (6-OHDA) rat model of PD. The results revealed that NAC enhanced DAn viability, as we found that it could restore dopamine transporter (DAT) levels compared to the untreated 6-OHDA group. Such findings were positively correlated with a significant amelioration in the motor outcomes of the 6-OHDA-treated animals, demonstrating that NAC may, somehow, be a modulator of PD degenerative mechanisms. Overall, we postulated a proof-of-concept milestone concerning the therapeutic application of NAC. Nevertheless, it is extremely important to understand the complexity of this drug and how its therapeutical properties interact with the cellular and molecular PD mechanisms.

## 1. Introduction

Of all existing neurological pathologies, Parkinson’s disease (PD) is considered the fastest neurodegenerative disorder, growing in prevalence, disability, and deaths [1]. The number of people living with PD in Europe has reached over one million, with 75,000 new cases occurring every year [2], a number that will double by 2030 due to an aging population [3]. PD is characterized by a broad spectrum of central motor impairments associated with a dysfunction of the somatomotor system [4,5,6]. These disturbances include uncontrollable resting tremors, bradykinesia (slowness of voluntary movements), muscular rigidity, and gait instability [7]. The manifestation of the motor symptoms is gradual and generally asymmetrically appears at the beginning, but bilaterally develops with time and the evolution of the disease, leading to an increase in motor disturbances and the patient’s quality of life reduction [8,9]. Additionally, the appearance and development of several non-motor symptoms are also linked to PD, substantially contributing to the disability and the uncertainty of their diagnosis [7,10]. Pathologically, PD is characterized by the gradual degeneration of midbrain dopaminergic neurons (DAn) localized in the ventral tier of the substantia nigra pars compacta (SNpc) within the mesostriatal/nigrostriatal pathway [11,12]. With the progression of the disease and consequent DAn degeneration, fewer cellular dopaminergic innervations are present in the striatum.

As a consequence, the production and release of dopamine (DA) is remarkably decreased, leading to alterations in cellular and synaptic mechanisms throughout the basal ganglia (BG) network [13]. This decrease in DA levels causes less inhibition of the activity of striatal neurons, allowing them to fire in an irregular way, thereby leading to disturbances in movement control [13]. Moreover, the pattern of SNpc cell loss appears to converge with a decrease in the level of expression of the dopamine transporter (DAT) [14]. Another important neuropathological hallmark of PD is the development and posterior accumulation of intracellular cytoplasmic Lewy bodies in the surviving DAn composed by an abnormal, post-translationally modified, and aggregated form of a presynaptic protein—alpha-synuclein (αSyn) [15,16]. Therefore, PD is no longer considered a single clinical disease, but rather a syndrome of multiple causes and manifestations [9]. Despite decades of research in this field, the cause of this disease has not yet been discovered and is unlikely to emerge [17]. It has become challenging to define its chronology and distinguish the molecular and cellular alterations occurring before and after DAn loss [18]. Despite these issues, multiple studies have significantly contributed to a better understanding of the several dysregulated mechanisms and pathways involved [19]. Oxidative stress, the generation of reactive oxygen species (ROS), mitochondrial dysfunction [20], neuroinflammation, excitotoxicity [21], altered proteolysis (proteasomal and lysosomal) [12], and the activation of apoptotic pathways [22] are some of the major contributors and mediators of DAn degeneration and PD progression.

Over the past century, efforts have been made to develop an effective therapy for Parkinson’s. Nevertheless, they were insufficient or a little skewed, as the currently available pharmacological and non-pharmacological strategies to treat PD only act on its symptomatic dimension, as an attempt to provide transient symptom relief to improve functional capacity [23,24]. The mainstay of therapies resides in either the replacement of deficient DA content or in stimulating dopamine receptors (mimicking the effects of DA) [25]. The existing treatment approaches have been failing by showing quite variable outcomes, either displaying benefits (during the first years after disease diagnosis) or leading to adverse effects (due to higher doses in medium-late stages of the disease), thereby being insufficient to tackle the multifactorial profile of PD [23]. Moreover, these cannot abrogate or counteract dopaminergic neuronal death as PD progresses. Given this, a major goal of PD research should go beyond the DA field by using or developing disease-modifying and neuroprotective strategies that can slow or stop the underlying neurodegenerative processes.

Drug repurposing is a powerful approach for identifying new applications for approved or investigational drugs outside the scope of the original medical indication [26]. In this context, N-acetylcysteine (NAC), an antioxidant drug with potential new tricks in nervous system regeneration, has been indicated as a promising modulator of PD-relevant mechanisms [27]. Firstly, as a potent thiol antioxidant, it can exert direct actions by functioning as a free radical scavenger of H_2_O_2_ and other ROS and semiquinones/quinones that derive from DA (usually associated with cell death) [28]. Furthermore, this compound also targets indirect events since it fortifies the action of a major endogenous antioxidant system by increasing the intracellular levels of cysteine and, consequently, the synthesis of glutathione (GSH) [27]. Due to these effects, NAC is often considered a precursor of GSH synthesis and a stimulator of the cytosolic enzymes involved in GSH regeneration [29]. The depletion of GSH in the brain has been broadly linked to oxidative stress and cellular damage, contributing to the neurodegenerative process of PD [30]. However, the use of GSH as a CNS therapeutic agent is restricted since it is poorly diffusible across the blood-brain barrier (BBB), which indicates that higher doses are required to reach satisfying levels [29]. In contrast, being a cell membrane–permeable form of cysteine, NAC can cross the BBB, with biologically relevant levels being achieved in human cerebrospinal fluid (CSF) [31]. Once in the brain, it can increase neuronal GSH content and subsequently incite pro-neurogenic and neuroprotective events [27]. A recent controlled and randomized clinical trial has demonstrated that intravenous administration of NAC boosted blood redox rations of GSH in PD patients, with the authors proposing that such an increase may compensate for the hypothesized deficient activity observed in the disease (NCT01427517) [32]. More recently, NAC administration was considered a promising supporter of DA neuronal viability and functionality, given its postulated ability to modulate DAT concentration or its binding [33,34]. Previous pre-clinical models of PD supported such findings. For instance, in a cell line study, Monti et al. showed that a pre-treatment with NAC protects midbrain DAn from rotenone exposure, increasing their survival [33]. In addition, NAC supplementation compensated for the expression of both PD and apoptosis-related genes in rats’ brains [35]. Recently, a report on a cellular model of PD demonstrated, for the first time, that NAC nanocarriers exhibited a more vital ability to protect cells from oxidative stress, increased iron levels, and lipid peroxidation [36]. Interestingly, Banaclocha and colleagues, using synaptic mitochondrial fractions isolated from mice, observed that NAC elicited an increase in mitochondrial complex I activity, which means that NAC applications can re-establish its loss after PD [37]. In a transgenic mouse overexpressing αSyn, NAC increased the densities of DAn terminals through a reduction of αSyn intracytoplasmic inclusions [38]. Thus, even being a promising approach, studies are warranted to support its ground-breaking potential and to widely understand how this approach could modulate motor symptomatology and cellular PD pathophysiological mechanisms.

Considering this, our initial work focused on characterizing an already described PD animal model induced by a unilateral injection of 6-OHDA into the dorsal striatum. Posteriorly, and to address the therapeutic relevance of NAC on functional and histological outcomes, fine motor coordination, limb placement and coordination, and dopaminergic networking were evaluated. From the results, we successfully validated the unilateral intrastriatal 6-OHDA rat model by showing mild impairments on specific behavioral tests. Considering the effects of the treatment pipeline, NAC monotherapy application displayed a proficient effect in reverting PD motor deficits and DAT levels, thereby indicating that our therapeutical strategy could potentiate DAn functional integrity preservation.

## 2. Materials and Methods

### 2.1. Animal Model

All animal experimentations were conducted after consent from the Portuguese national authority for animal research, Direção Geral de Alimentação e Veterinária (ID: DGAV005453, Lisbon, Portugal), and in accordance with the local regulations on animal care and experimentation (European Union Directive 2010/63/EU). All of the surgical procedures, namely from establishing the 6-hydroxydopamine (6-OHDA) PD model to administering NAC, were accomplished as previously described [34,39]. Eight-week-old Wistar-Han male rats (with ≈ 300 g; Charles River, Barcelona, Spain, http://www.criver.com/, accessed on 3 January 2023) were housed in pairs, in appropriate cages with adequate refinement, and maintained under standard controlled environment: room temperature at 22–24 °C and 55% humidity, 12-h light/dark cycles, regular rodents’ chow and tap water *ad libitum*. To reduce the stress induced by the surgical procedures, the animals were handled a week before the start of the experimental protocol.

### 2.2. Intrastriatal 6-Hydroxydopamine (6-OHDA)-Induced Lesions and Treatment Application

**Figure 1:**
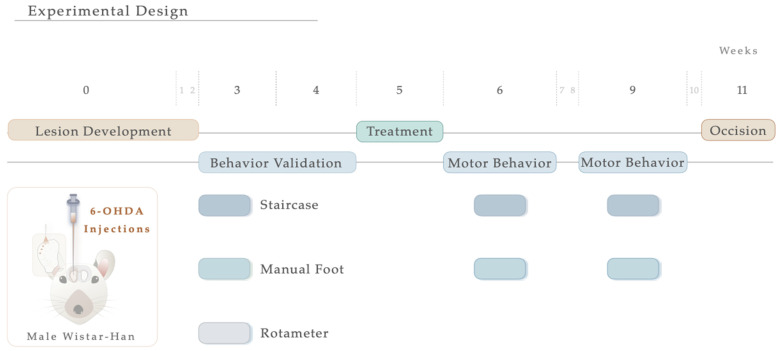
Experimental timeline. Schematic illustration showing the sequence of events of the study, including 6-OHDA intrastriatal lesion induction, NAC oral administration, and motor behavior analysis.

For the induction of the PD model, the animals were initially intraperitoneally (IP) anesthetized with a mixture of ketamine-medetomidine (75 mg/kg; 0.5 mg/kg) and later positioned on a stereotaxic frame (Stoelting, Wood Dale, IL, USA). Lesions were induced by a unilateral injection, using a 30-gauge needle Hamilton syringe (Hamilton, Bonaduz, Switzerland https://www.hamiltoncompany.com, accessed on 3 January 2023), with either vehicle (Sham group, n = 9) or 6-OHDA (Sigma, St. Louis, MO, USA, n = 20) directly into 4 distinct sites of the right striatum (coordinates related to Bregma: AP: +1.3; ML: −2.6; DV: −5.0; AP: +0.4; ML: −3.0; DV: −5.0; AP: −0.4; ML: −4.2; DV: −5.0; AP: −1.3; ML: −4.5; DV: −5.0; according to Paxinos and Watson brain atlas [40] and Kirik et al. [39]). At a rate of 1.0 μL/min, sham animals received 4 μL of 0.2 mg/mL of ascorbic acid in 0.9% of NaCl, and 6-OHDA animals were injected with 4 μL of 6-OHDA hydrochloride (7 μg) with 0.2 mg/mL of ascorbic acid in 0.9% of NaCl. Subsequently, the needle was left in place for 2 min to avoid backflow. Ultimately, the animals were sutured and injected with 100 μL of anti-sedation (Orion Pharma, Espoo, Finland) to recover from the surgery. Animals were randomly assigned to each group, keeping researchers blind to their choice, and the contralateral side (no injection) acted as a control. For model validation, motor behavioral assessments were performed three weeks following the surgical procedure (Figure 1). Five weeks after the 6-OHDA injection and following blind conditions, animals received N-Acetylcysteine. NAC tablets (Prozis, Machico, Portugal) were crushed in water and administered by oral gavage (single dose administration, 123.5 mg/kg, human equivalent dosage to 1200 mg/day following the formula for dose translation [41]). This route of administration was selected since it replicates the human therapeutic setting [34]. As a result, the animals were assembled into 3 distinct groups: (1) Sham (n = 9, injected with sterile saline); (2) 6-OHDA (n = 11, injected with the vehicle—saline); (3) NAC (n = 9). One and four weeks after treatment, the animals’ motor performance was assessed. The experimental timeline, including surgical procedures and behavioral evaluation, is graphically outlined in Figure 1.

### 2.3. Behavioral Testing Paradigms

The motor behavioral assessments of the animals were made first to characterize the 6-OHDA PD model and, secondly, to analyze the impact of treatment administration.

#### 2.3.1. Skilled Paw-Reaching Test

To evaluate fine motor control during skilled forelimb use, the staircase test was performed as previously described by Montoya et al. [42]. Herein, the rats’ ability to independently reach, grasp, and retrieve food pellets from the steps of double staircase boxes (80300, Campden Instruments Ltd., Loughborough, Leicestershire, UK) was assessed. Briefly, the test apparatus consisted of a clear chamber with a hinged cover (Campden Instruments, Lafayette, LA, USA) connected to a narrower compartment with a central raised platform running along its length. A removable double staircase was inserted at the end of the box, containing 7 steps on each side. Each step of the 7-step staircase comprised a small 3-mm deep well where food pellets were placed. Before the test, five pellets were placed into each well of the double staircase apparatus. Since food deprivation was required, animals were put to starvation one day before the beginning of the test. A two-day training session occurred, where on day one, the rats were allowed to become familiarized with the chambers for 5 min, and on day two, the animals were placed for 10 min in the box and presented with sugar pellets on each well and along the central plinth. During 5 consecutive days, animals were maintained inside the chamber and bilaterally exposed to food pellets, having 15 min to reach, retrieve, and eat the pellets in the wells. After each test period, the staircase was detached, and the number of pellets taken and eaten per side was counted. All sessions were performed at the same time, during the daytime. Finally, in the last two days of testing, animals were exposed to a forced-choice task (FC), in which the animals were forced to choose one of the steps-side of the double staircase (left or right), thus allowing for the measurement of the dimension of impairment on the affected side. For each session, the forelimb function was measured as the success rate; i.e., calculating the percentage of total pellets successfully retrieved. Results were plotted for each group and each testing day.

#### 2.3.2. Manual Foot Misplacement Corridor

**Figure 2:**
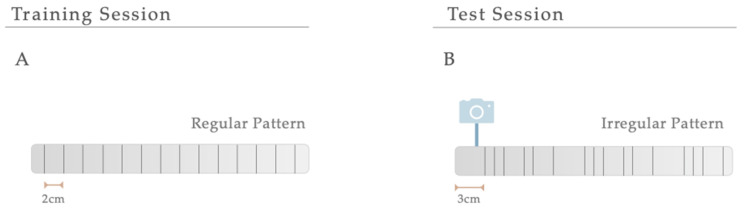
Serial Patterns. The difficulty of the manual foot misplacement corridor test was changed by varying the spacing between bars (across the entire apparatus), producing two different serial patterns. (**A**) A regular pattern was used for a training session, and (**B**) an irregular one was created for the test sessions.

In the manual foot misplacement corridor test, the animals had to walk on a 1-m long horizontal runway with round metal rungs and randomly assigned gaps. This motor behavioral test sets accuracy constraints on the locomotive performance, since the animals must precisely place their fore- and hindlimbs on the bars [43]. Before performing the test, the equipment and video recording system were set. The ladder rung apparatus was composed of side walls made of clear Plexiglas and metal bars, which could be inserted to create a floor with a minimum distance of 1 cm between the rungs, allowing the establishment of different patterns. Since animals were familiarized with training, the elevation of the apparatus was unlikely to cause anxiety. The alley’s width was adjusted to the animal’s size, so it was about 1 cm wider than the animal to avoid it from turning around. The task’s difficulty was changed by varying the spacing between metal bars (across the entire apparatus), producing two different serial patterns (Figure 2). Over a two-day training session, a regular pattern was used to familiarize the animals with the apparatus and anticipate the rungs’ position. An irregular pattern was used during the test sessions. For the regular arrangement, the bars were spaced at 2-cm intervals. For the irregular pattern, the distance of the bars systematically varied from 1 to 3 cm. Regarding the training session, all animals were instructed to cross the ladder from the neutral cage to reach their home cage (functioning as positive reinforcement for walking) and crossed it always in the same direction. For two consecutive days, rats were trained to cross the corridor in five trials. Upon this, and on two additional days, testing sessions were performed. Each test session consisted of three trials during which the animals’ performance was video recorded from a lateral perspective. With the help of a mirror, the video recording camera (Sony FDR-AX33 digital 4K) was positioned at a slight ventral angle, so that both sides and paw positions could be simultaneously recorded from a ventral view. For quantitative and qualitative analysis, the videos were analyzed frame-by-frame, in slow motion using the iMovie Editor Software (version 10.2, Apple). A 6-category rating foot fault scoring system previously developed was used as a qualitative analysis to determine the type of forelimb and hindlimb placement on the bars [43]. Each step during a pass down the ladder was scored. However, the first two initiation steps and the last two final steps were not included. The last step before a pause and the first step after a pause were also excluded from scoring. Each step was scored according to the limb position quality and errors in placement accuracy. Concerning quantitative analysis, this was based on the number of errors in each crossing and latency (average time needed to cross the entire length of the ladder task). Errors were determined based on the scoring system mentioned. An error was defined as each limb placement that received a score of 0, 1, or 2 points. During the experiment, the animals performed a minimum of 9 and a maximum of 14 steps.

#### 2.3.3. Apomorphine Turning Behavior

Drug-induced contralateral rotational behavior was performed using apomorphine, a potent DA agonist capable of inducing hyperstimulation of DA receptors in the lesioned striatum [44]. This test was applied to evaluate the extent of DA depletion on the dopaminergic denervation, validating the model and thus selecting the animals that were truly lesioned upon 6-OHDA injections. As earlier described [45], all animals received 0.05 mg/kg of apomorphine hydrochloride (Sigma, St. Louis, MO, USA) solution dissolved in 1% of ascorbic acid in 0.9% of NaCl, which was subcutaneously injected in the neck. Afterward, they were directly retained to an automated circular testing cage (rotameter; MED-RSS, Med Associates, Georgia Regional Industrial Park, 166 Industrial Park Road, USA), and the rotational score was digitally recorded over 30 min. The rotational outcome was acquired by subtracting the total number of ipsilateral turns from the total number of contralateral rotations. Data are expressed as net contralateral rotations. The rotameter test was only used to validate the PD model since the frequent use of apomorphine could lead to an overstimulation of the DA system, leading to an inadequate interpretation of treatments effects on the functional outcomes [46].

### 2.4. Histological Analysis

Ten weeks after the development of the 6-OHDA PD model and subsequent treatment and behavioral evaluation, the animals were euthanized with sodium pentobarbital (Eutasil, 60 mg/kg, i.p., Ceva Saúde Animal, Portugal) and transcardially perfused with saline (0.9% NaCl) and 4% paraformaldehyde (PFA) in 0.1% 1 × PBS. Afterward, the brains were collected and post-fixed in 4% PFA for 24 h at 4 °C. The brains were then transferred to a 30% sucrose (with PBS and 0.1% azide) solution the next day and were maintained at 4 °C. For histological procedures and analysis, striatal and mesencephalon coronal sections (comprising SNpc) 40μm thick were obtained using a vibratome (Leica, VT1000S, Wetzlar, Germany) and processed as free-floating sections.

#### 2.4.1. Tyrosine Hydroxylase (TH) Immunohistochemistry

Six series of consecutive sections of the striatum and SNpc (40μm thick) were selected for free-floating. Briefly, slices were immersed for 20 min in 1 M PBS with 3% H_2_O_2_ to inhibit endogenous peroxidase activity. Then, the sections were washed with 1 M PBS (10 min) and then permeabilized with 0.1% PBS-Triton (PBS-T) for ten additional minutes. Nonspecific binding was blocked for 2 h with 10% fetal calf serum (FCS; Thermo Fisher Scientific Life Sciences, Waltham, MA, USA) in 1 M PBS. Then, slices were incubated overnight at 4 °C, with mouse tyrosine hydroxylase (TH) primary antibody (1:1000; Merck Millipore, Boston, MA, USA) diluted in 1 M PBS with 2% FCS. After rinsing the sections 3 consecutive times with 0.3% PBS-T (10 min each), they were incubated for 30 min at room temperature with a biotinylated secondary antibody (ThermoFisher Scientific, Waltham, MA, USA, TP-125-BN) and were, posteriorly, washed 3 times in PBS with 0.3% Triton, for 10 min. After that, a 30-min incubation was carried out with a streptavidin-peroxidase solution (ThermoFisher Scientific, TB-125-HR). Following this, the samples were washed 3 times in 0.1% PBS-T, washed 1 time in 1 M PBS (10 min each), and 0.5 M Tris-HCl. Antigen detection was accomplished using 3,3′-diaminobenzidine tetrahydrochloride (DAB; D5905, Sigma) (25 mg DAB in 50 mL Tris–HCl 0.05 M with 12.5 μL H_2_O_2_, pH 7.6) with 12.5 mL of H_2_O_2_, and color reaction was stopped after 1–1.5 min. The slices were then mounted on superfrost^®^ Plus slides (ref. 631-0108P; VWR, Galdenaaksebaan, Leuven, Belgium) using entellan (Merck Millipore, Boston, MA, USA) and left to air-dry in the dark for 24 h at RT. To ensure a representative sampling between animals, 6 TH-labelled slices spanning the entire mesencephalon and 6 TH-immunostained prosencephalon sections were selected.

#### 2.4.2. Stereological Quantification and Striatal-Fiber TH^+^ labeling

The estimation of the TH-positive cells’ preservation was made by counting the total number of DA neurons in the SNpc, using a bright-field microscope (BX51, Olympus, Center Valley, PA, USA) equipped with a digital camera (PixeLINK PL-A622, CANIMPEX Enterprises Ltd., Halifax, NS, Canada). Four TH-labeled slices along the anterior-posterior axis of the mesencephalon were selected, comprising all the portions of the SN. With the help of Visiopharm software (V2.12.3.0; Visiopharm, Hørsholm, Denmark), the boundaries of the SNpc area were drawn. The delineation of this region was performed based on the anatomical identification of reference points. The counting of total TH-immunopositive cells in the SNpc area was performed on both hemispheres (40× magnification). Data are represented as the percentage (%) of remaining TH-positive cells on the injected side, compared to the control (intact) side. TH^+^ labeling in the dorsal striatum was measured by densitometry. For this purpose, 4 TH immunostained prosencephalon sections, representing the coordinates of injection sites within the striatum, were chosen and photographed (with an Olympus Cx31 light microscope equipped with a DP71 camera, Shinjuku, Tokyo, Japan). Photographs were converted to greyscale using the Image J program (2.9.0/1.53 version; National Institute of Health, Bethesda, MD, USA) and analyzed for the grey intensity of TH^+^ labeling after calibrating the software program. Striatum TH^+^ labeling values were determined on both brain hemispheres, following the program instructions. Using the ROI manager tool, eight distinct regions were drawn, containing the contralateral striatum, ipsilateral striatum, background ipsilateral, and contralateral (three different areas of the corpus callosum, each). These background measurements were used as an internal control for normalizing nonspecific signal background. TH-positive striatal-fiber densities were determined by calculating the optical density (O.D.) difference between the ipsilateral lesioned striatum side with the intact one. The data are expressed as percentages (%) of the contralateral striatum (undamaged side). All analyses were performed under blind conditions.

#### 2.4.3. Immunofluorescence Staining, Image Acquisition, and Analysis

Four free-floating coronal sections of the striatum were obtained and processed as previously described. Double immunofluorescence techniques were used to determine the presence of DAT and TH-positive fibers in the striatum using the procedure described below. Antigen retrieval was performed for 20 min using heated citrate buffer (90 °C, pH 6.0). As a first approach, slides were washed 3 times with 0.5% PBS-T (Triton X-100), followed by a subsequent permeabilization step using 0.5% PBS-T for 10 min. Afterward, the sections were washed 3 times with 0.5% PBS-T and incubated in blocking serum (10% normal goat serum (NGS; Sigma-Aldrich, St. Louis, MO, USA) plus 0.5% PBS-T for 30 min. Later, the primary antibodies TH (mouse, 1:1000, Millipore (USA)) and DAT (rabbit, 1:250, Abcam (UK)) diluted in 3% NGS (Sigma-Aldrich, St. Louis, MO, USA) and 1 × PBS were incubated overnight at 4 °C. The next day, sections were washed 3 times in 1 × PBS, and secondary antibodies were raised on an anti-mouse or anti-rabbit (AlexaFluor 488 and 594, ThermoFisher Scientific, Waltham, MA, USA) prepared solution in 1 × PBS incubated for 2 h at RT on an orbital shaking platform set at 120 rpm. After this, slices were washed 3 times in 1 × PBS and were counterstained with diamidino-2-phenylindole-dihydrochloride (DAPI; 1:1000; ThermoFisher). Slices were mounted on superfrost^®^ Plus slides (ref. 631-0108P; VWR, Galdenaaksebaan, Leuven, Belgium) and coverslipped with Permafluor (ThermoFisher, Waltham, MA, USA). Imaging workflows were developed to scan striatal areas. Photomicrographs from the immunofluorescence stains were acquired with a confocal laser scanning microscope (Olympus FV3000, Shinjuku, Tokyo, Japan) and using the software FV10-ASW 2.0c (Olympus). To standardize image acquisition and minimize inter-sample variation, the entire sections were imaged at 2× magnification using DAPI and TH markers, enabling the creation of imaging maps and the correct detection of lesioned and unlesioned hemispheres. All photos were taken using lasers 405 (Blue), 488 (Green), and 594 (Red). Striatal images of both ipsi and the contralateral side, encompassing four different regions of the dorsal-ventral axis, were acquired. Double fluorescent stainings were analyzed using a semi-automated workflow developed on FIJI (NIH, USA). Images were first processed to produce projections along the *Z*-axis and an automatic intensity-based thresholding was applied to separate foreground pixels from background pixels. Accordingly, the mean grey value of DAT was determined in both ipsi and the contralateral side, and the results were expressed as the percentage (%) of fluorescence intensity on the injected side, compared to the control side. Additional representative images of DAT immunofluorescence in the striatum were acquired using the fluorescence microscope (Olympus Widefield Upright Microscope BX61, Shinjuku, Tokyo, Japan). A minimum of two coronal sections per animal were assessed, and brain sections that were cracked, folded, or washed off during the immunostaining procedure were excluded from the analysis. For all the studies, researchers were blinded to the treatment groups.

### 2.5. Statistical Analysis

Statistical analysis was carried out using the SPSS Statistic Program (version 26; IBM Co., Armonk, NY, USA) and GraphPad Prism 8 software, specifically for graphics design (La Jolla, USA). Normality assumption was evaluated for all continuous variables using the Shapiro-Wilks statistical test, considering the respective measures of skewness and kurtosis and visualizing normal histogram distributions. Levene’s and Mauchly’s tests appraised the homogeneity of variances and sphericity, respectively, and assumed when the *p*-value ≥ 0.05. Student’s *t*-tests for independent samples or Mann-Whitney *U* tests (data presented as mean + IQR) were carried out to compare means between two groups. For lengthwise comparisons of mean differences, both two-way ANOVA analysis and mixed-design factorial ANOVA were applied. To further evaluate the nature of the differences between groups, multiple comparison post hoc tests were performed, employing either Tukey’s HSD post hoc test or Bonferroni’s. All tests were performed with a 95% confidence interval. Data were acknowledged as statistically significant if the *p*-value ≤ 0.05. Suitable effect sizes for each test were calculated (ANOVA: eta-square partial (η^2^_partial_); *t*-test: Cohen’s d (d); Mann Whitney *U*-test: rank-biserial correlation(r)). All graphs are represented with mean values ± SEM (standard error of the mean).

## 3. Results

### 3.1. Intrastriatal 6-OHDA PD Model

#### Motor Behavioral Characterization and Phenotypic Validation

**Figure 3:**
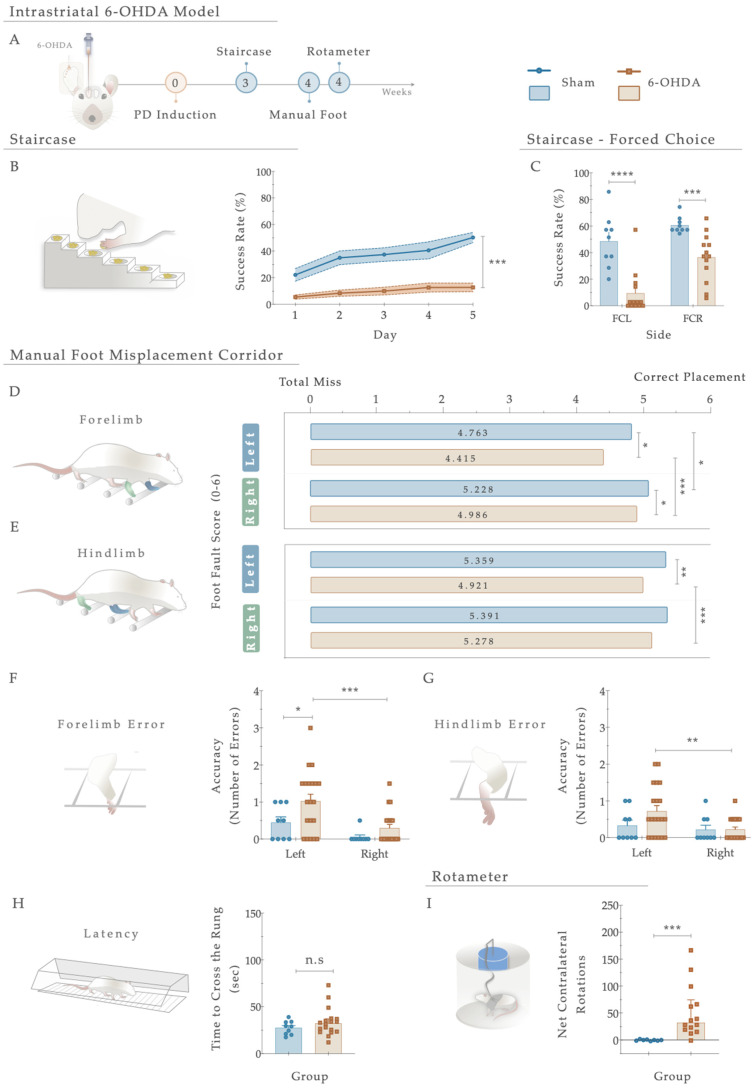
(**A**) Phenotypic characterization of the 6-OHDA intrastriatal PD model. Three weeks after 6-OHDA exposure, through the staircase test, motor deficits were observed with a (**B**) significant reduction in fine motor coordination of the 6-OHDA animals. Performing a forced-choice task, a significant impairment on both the affected side (left side) and the right side was perceived when compared to the control group (Sham; (**C**)). The manual foot misplacement corridor test assessed skilled walking (**D**–**H**). In the foot fault score, regarding 6-OHDA animals, the average stepping score for both limbs was significantly different from the Sham group (**D**,**E**), and statistical differences were observed in ipsilateral versus contralateral placement (**D**,**E**). Considering placement accuracy, differences between the Sham and 6-OHDA groups were only obtained in the left forelimb (**F**). In contrast, among 6-OHDA animals, an increased number of contralateral errors was observed in both fore- and hindlimbs (**F**,**G**). Moreover, group differences in latency to cross the apparatus (**H**) were subtle but not significant (*p* = 0.348). In the apomorphine turning behavioral test, the injection of 6-OHDA in the striatum led to a significantly higher number of net-contralateral rotations when compared with the Sham group (**I**), demonstrating that animals’ dopaminergic integrity was affected. Data are presented as mean ± SEM and mean ± IQR. For the tests: Sham: n = 8–9; 6-OHDA: n = 13–20. * *p* < 0.05, ** *p* < 0.01, *** *p*< 0.001, **** *p*< 0.0001. Abbreviations: FCR, forced-choice task at the right side; FCL, forced-choice task on the left side; n.s: nonsignificant; sec, seconds; 6-OHDA, 6-Hydroxydopamine.

Three weeks after the 6-OHDA injections into the right striatum (Figure 3A), the general motor performance of the animals was assessed to behaviorally characterize the development of PD-like motor deficits. Starting with the staircase test (performed to address fine motor coordination), statistical analysis revealed that compared to the Sham animals, the 6-OHDA-injected animals displayed a significant inability to successfully grasp, retrieve, and eat the pellets placed on the staircase apparatus (Figure 3B). As such, the 6-OHDA injections clearly affected forelimb function, developing severe motor impairments in the lesioned group compared to the control group (Sham; F _(4, 108)_ = 5.194, *p* < 0.001, η^2^_partial_ = 0.161; Figure 3B). Furthermore, concerning the forced choice task (where animals were forced to choose one of the step sides), the behavior of the 6-OHDA animals was significantly impaired when directly compared to the control group (Sham; right side (FCR): t _(20)_ = 4.487; *p* = 0.0004, Cohen’s d = 1.801; and left side (FCL): t _(20)_ = 5.054; *p* < 0.0001, Cohen’s d = 2.145. Figure 3C).

Regarding skilled walking, the placement of the fore- and hindlimbs on the ladder rung walking task were first assessed with a foot fault score (Figure 3D,E). The average limb scores of the irregular pattern were separately analyzed, with the same scoring system being applied in both forelimbs and hindlimbs, and six indicating a correct placement. Forelimb scores were subjected to a two-way ANOVA. From the results, the between-subjects effect was significant (i.e., lesion factor: F _(1,26)_ = 14.073, *p* = 0.001, η^2^_partial_ = 0.351) and also for the within-subjects effect (i.e., side (left or right): F _(1, 26)_ = 22.02, *p* < 0.0001, η^2^_partial_ = 0.459). However, the interaction between all the factors was not significantly achieved (F _(1,26)_ = 0.006, *p* = 0.937, η^2^_partial_ = 0.051). In forelimb performance, the average score on both sides was superior in the controls than in the 6-OHDA animals (as visualized in Figure 3D), demonstrating that the forelimbs of lesioned animals have an increased probability of failing. Indeed, all 6-OHDA animals showed functional impairments in both contralateral and ipsilateral forelimb performance compared to the Sham (contra: *p* = 0.026, ipsi: *p* = 0.035; Figure 3D). Moreover, a significant difference was also observed between the ipsi- and contralateral forelimbs of 6-OHDA animals (*p* = 0.0003, Figure 3E). Among hindlimbs, statistical analysis revealed an effect for the factor lesion (F _(1,27)_ = 7.207, *p* = 0.012, η^2^_partial_ = 0.211) and factor side (F _(1, 27)_ = 5.81, *p* = 0.023, η^2^_partial_ = 0.117), but no interaction between these factors (F _(1,27)_ = 4.030, *p* = 0.055, η^2^_partial_ = 0.130; Figure 3E). After lesioning, in 6-OHDA animals’, statistical differences in the contralateral side were reached compared to the vehicle group (Sham; *p* = 0.0030, Figure 3E). Similarly, among the 6-OHDA rats, significance was observed in contralateral *versus* ipsilateral hindlimbs placement (*p* = 0.001), reflecting, once again, that an impaired foot placement was established after 6-OHDA injections (Figure 3E).

Concerning the number of errors (an indicator of placement accuracy; Figure 3F,G), statistical analysis revealed an effect on the lesion (F _(1, 27)_ = 5.808, *p* = 0.023, η^2^_partial_ = 0.177) and side (F _(1, 27)_ = 11.47, *p* = 0.002, η^2^_partial_ = 0.298) conditions on the forelimbs, but no effect on side-by-lesion interactions (F _(1, 27)_ = 1.044, *p* = 0.316, η^2^_partial_ = 0.037). Compared to the Sham group, an increase in the number of errors on the left side of the 6-OHDA group was observed (*p* = 0.036, Figure 3F). In addition, differences between contra- and ipsilateral errors in the 6-OHDA group were distinguished (*p* = 0.0010, Figure 3F). Notably, when we assessed hindlimb errors, only a side effect was observed (Side: F _(1, 27)_ = 7.041, *p* = 0.013, η^2^_partial_ = 0.207; Lesion: F _(1, 27)_ = 1.518, *p* = 0.228, η^2^_partial_ = 0.053; Interaction: F _(1, 27)_ = 2.851, *p* = 0.103, η^2^_partial_ = 0.096). Accordingly, the number of hindlimb contralateral errors increased exclusively in the animals injected with 6-OHDA (*p* = 0.001; Figure 3G). In general, the number of errors in forelimbs among animals was higher than in hindlimbs. Furthermore, group differences in time measurements were subtle (Figure 3H) but not significantly distinctive between the controls and 6-OHDA animals (U = 62.00, *p* = 0.348, r = 0.036).

Finally, the rotameter test was performed to address the integrity level of the dopaminergic system, and hence, choose the truly injured animals (Figure 3I). After apomorphine administration, statistical examination revealed an intense turning behavior in the 6-OHDA-injected animals compared with the Sham group (U = 6.0, *p* = 0.0002, r = 0.557), demonstrating a greater lesion extent was achieved.

### 3.2. NAC Attenuated Paw Reaching Motor Coordination Deficits in 6-OHDA PD Animals

**Figure 4:**
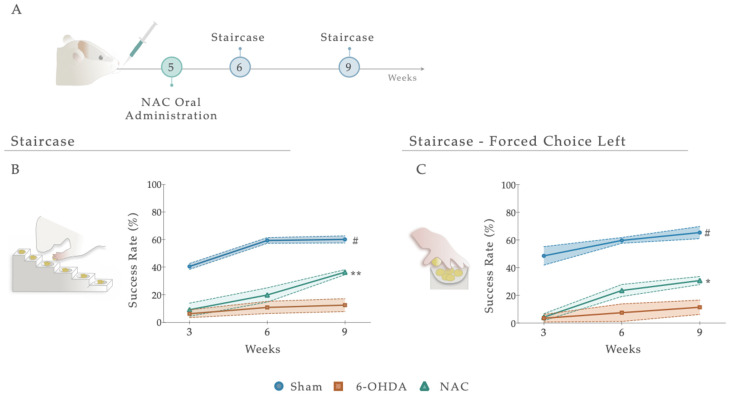
(**A**) Impact of NAC administration on skilled motor performance, 1 and 4 weeks after treatment administration. Under a paw-reaching test, all treated animals demonstrated a significant amelioration (*p* < 0.001) of forelimb coordination, with a higher success rate of eaten pellets (%) when compared to the untreated group, 6-OHDA (**B**). When rats were submitted to the (**C**) paw-reaching forced performance task, statistical analysis revealed significant effects between treated and 6-OHDA untreated animals. Data are presented as mean ± S.E.M. For the tests: Sham n = 8–9; 6-OHDA n = 8; NAC n = 8–6. * *p* < 0.05, ** *p* < 0.01; Sham animals were statistically different from all 6-OHDA injured animals: # *p* ≤ 0.0001. Abbreviations: NAC. N-Acetylcysteine; 6-OHDA, 6-Hydroxidopamine.

Following treatment administration, after 1 and 4 weeks, the staircase test was employed to determine the therapeutic impact of NAC on forelimb use and the fine motor coordination of 6-OHDA animals (Figure 4A and Figure S1). Statistical analysis revealed that NAC-treated animals displayed a significant amelioration (*p* < 0.01) in forelimb coordination, with a noteworthy increase in the success rate of eaten pellets when compared to the untreated group (6-OHDA; Figure 4B). Indeed, the statistical report exposed a significant between-subjects effect (i.e., factor treatment; F _(2, 19)_ = 59.48, *p* < 0.0001, η^2^_partial_ = 0.856), within-subjects effect (i.e., factor time; F _(2, 38)_ = 42.00, *p* < 0.0001, η^2^_partial_ = 0.689), and an interaction between these factors (Figure 4A; F _(4, 38)_ = 7.141, *p* = 0.0002, η^2^_partial_ = 0.429). This piece of evidence was also observed under a paw reaching forced task on the (left) affected side (FCL), where statistical analysis revealed effects for the factor treatment (F _(2, 22)_ = 54.17, *p* < 0.0001, η^2^_partial_ = 0.831), time (F _(2, 44)_ = 21.80, *p* < 0.0001, η^2^_partial_ = 0.498), but no interaction between them (F _(4, 44)_ = 2.287, *p* = 0.075, η^2^_partial_ = 0.172; Figure 4C). Comparing the NAC-treated animals with the untreated group (6-OHDA), post hoc analysis revealed that the administration of the NAC led to a significant amelioration of animals’ motor performance in the affected side only at week 9 (*p* < 0.05, Figure 4C). In both assays, the skilled performance of the left forelimb of Sham animals was statistically different from all other groups, with a predictable higher motor function (Figure 4B,C).

### 3.3. Effects of NAC Administration on Locomotor Function and Precision of 6-OHDA PD Animals

**Figure 5:**
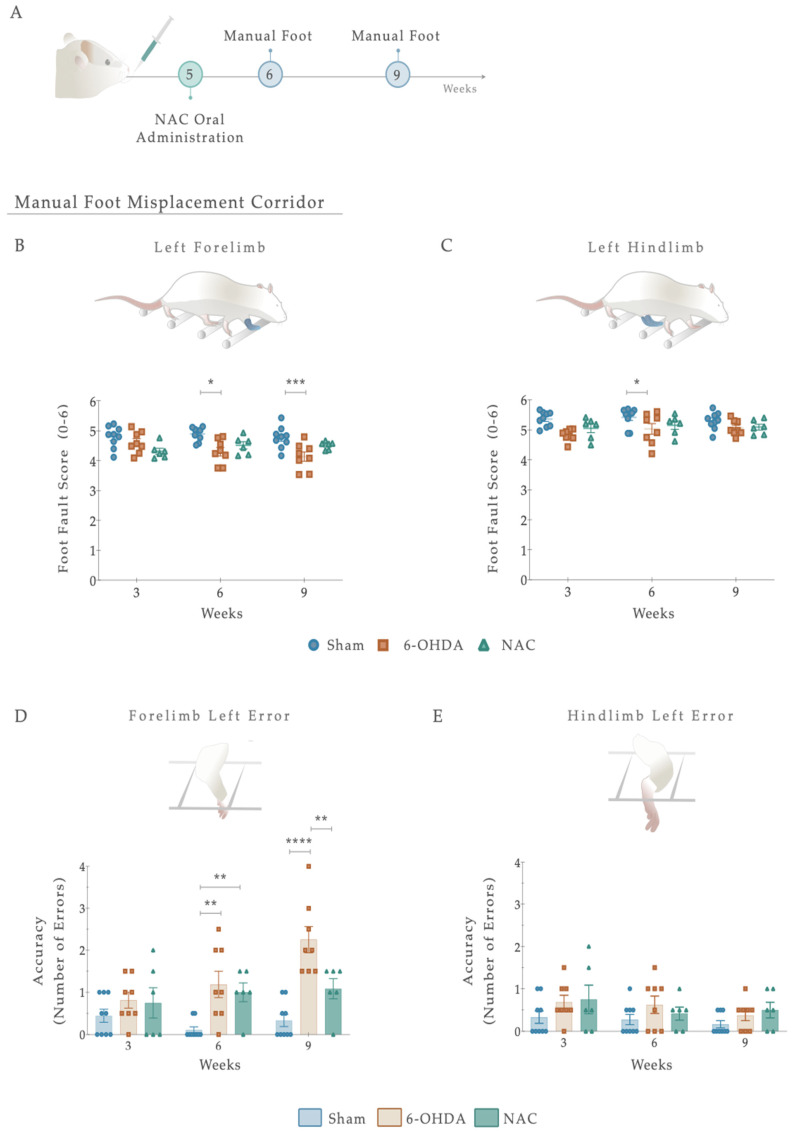
(**A**) Beneficial effects of NAC intervention on locomotor function, 1 and 4 weeks after treatment administration. Overall, 6-OHDA-lesioned animals showed impairments in left limb placement 6 and 9 weeks after intrastriatal injections (**B**,**C**). No signs of amelioration of these deficits were observed upon NAC administration (**B**,**C**). Regarding placement accuracy (number of errors), 6-OHDA animals exhibited an increase in the number of errors throughout the treatment weeks compared to the control group (**D**). Remarkably, in NAC-treated rats, the left footfall pattern of forelimbs significantly improved in this task in week 9 (**D**). In contrast, all groups’ error counts on the contralateral hindlimb were evenly distributed (**E**). Data are presented as mean ± S.E.M. For the tests: Sham n = 9; 6-OHDA n = 8; NAC n = 6. * *p* < 0.05, ** *p* < 0.01, *** *p* < 0.001, **** *p* < 0.0001. Abbreviations: NAC. N-Acetylcysteine; n.s: nonsignificant; 6-OHDA, 6-Hydroxidopamine.

To test whether NAC administration promotes the recovery of spontaneous and voluntary locomotion and motor coordination following 6-OHDA injury, the manual foot misplacement test was then used (Figure 5A). As a primary result, statistical analysis revealed that the limbs on the ipsilateral (right) side demonstrated minimal changes in the foot fault scores, 1 and 4 weeks after treatment administration (Figure S2). Regarding the contralateral (left) side, although no differences were observed for within-subjects effect (i.e., factor time F _(2, 40)_ = 0.800, *p* = 0.456, η^2^_partial_ = 0.038), fine paw placement of the forelimb displayed significance for between-subjects effect (i.e., factor treatment: F _(2, 20)_ = 9.559, *p* = 0.001, η^2^_partial_ = 0.489) and on the interaction between factors (F _(4,40)_ = 2.707, *p* = 0.044, η^2^_partial_ = 0.213; Figure 5A). Our results showed that in the 6-OHDA group, foot-fault scores of the forelimbs remained significantly lower 4 weeks after treatment compared to the Sham group (*p* < 0.001; Figure 5B), suggesting a persistent deficit in fine motor skills. Importantly, and in contrast to the 6-OHDA animals, NAC-treated rats did not have significant differences in forelimb correct placement (Figure 5A). Similar results were found on the posterior limb, as only an effect of the factor treatment was revealed (Treatment: F _(2, 20)_ = 7.854, *p* = 0.003; η^2^_partial_ = 0.440; Time: F _(2, 40)_ = 0.994, *p* = 0.379, η^2^_partial_ = 0.047; Interaction: F _(4, 40)_ = 0.574, *p* = 0.683, η^2^_partial_ = 0.054; Figure 5C). Analysis of left hindlimb paw placement revealed that 6-OHDA animals had decreased step scores compared to the vehicle-treated group (Sham; *p* < 0.05; Figure 5C). However, no significant differences in recovery were observed between NAC and 6-OHDA animals (*p* = 0.485; Figure 5C).

We next sought to determine whether foot placement accuracy (number of errors) could be improved upon NAC monotherapy (Figure 5D,E). For that, the number of errors in each crossing was counted. From such analysis, the placement accuracy of ipsilateral limbs did not differ between the groups (Figure S2). Nevertheless, when considering the lesioned side, group differences were revealed in forelimb errors with an effect of factor treatment (F _(2, 20)_ = 18.47, *p* < 0.0001, η^2^_partial_ = 0.649), factor time (F _(2, 40)_ = 5.237, *p* = 0.01, η^2^_partial_ = 0.208), and the interaction between the two (F _(4, 40)_ = 3.844, *p* = 0.01, η^2^_partial_ = 0.278; Figure 5D). Following treatment exposure, the 6-OHDA group showed a gradual increase in the number of errors, with post hoc analysis displaying a significant difference when compared with the Sham animals (*p* < 0.0001, Figure 5D). Interestingly, in NAC-treated rats, the left footfall pattern was steady and consistent throughout the weeks, becoming significant from lesioned animals at week 4 (*p* = 0.003, Figure 5C). In contrast, all groups’ error counts on the contralateral hind paw were evenly distributed. The analysis conducted failed to reveal significant effects (Treatment: F _(2, 20)_ = 3.097, *p* = 0.067, η^2^_partial_ = 0.236; Time: F _(2, 40)_ = 1.666, *p* = 0.2018, η^2^_partial_ = 0.077; Interaction: F _(4, 40)_ = 0.333, *p* = 0.854, η^2^_partial_ = 0.032; Figure 5E). Additionally, we observed no significant effects of NAC treatment on latency to cross the apparatus (Figure S2).

### 3.4. NAC Monotherapy Modulates Dopaminergic Histological Deficits

**Figure 6:**
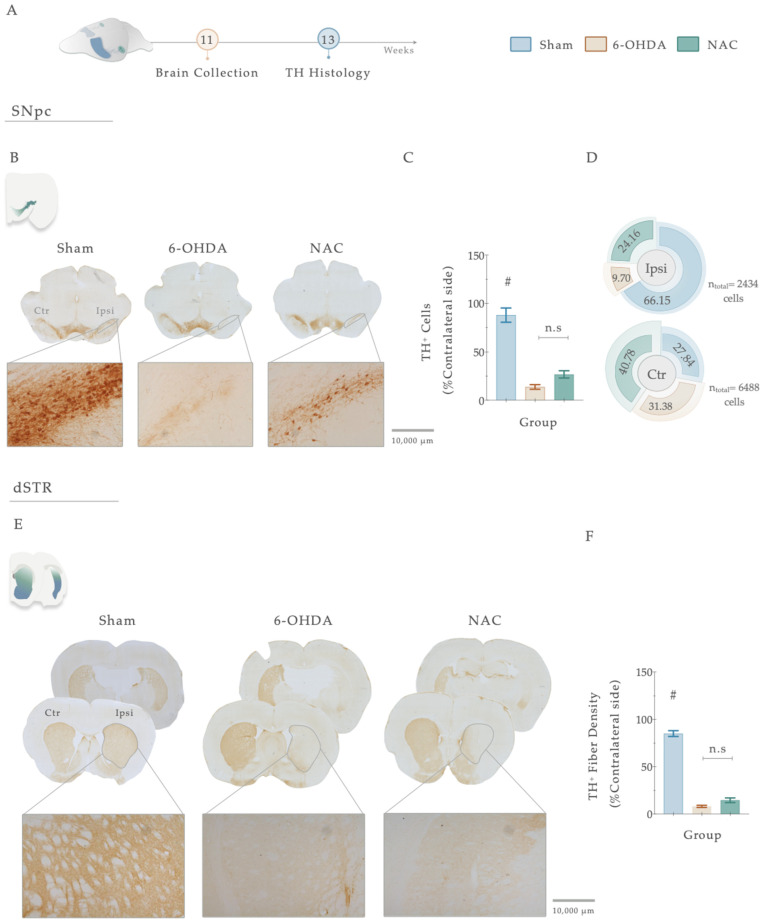
(**A**) Histological assessment of NAC therapeutical administration in the central dopaminergic network. Representative image of SNpc and dorsal striatum analyzed and brightfield photomicrographs of the selected brain sections stained for TH (**B**,**E**). Concerning the SNpc, all animals injected with 6-OHDA presented a significant loss of TH^+^ cells when compared to the Sham group (**C**). Pie chart illustrating the percentage of TH^+^ cells in the SNpc, on both ipsilateral and contralateral sides (**D**). TH^+^ neurons count revealed a general trend towards a higher distribution of these cells in the ipsilateral lesioned side of NAC-treated animals than in the 6-OHDA group (**D**). For the dorsal striatum (**E**), although 6-OHDA animals presented a marked reduction in TH^+^ labeling, treatment intervention with NAC did not have an impact on striatal histological deficits (*p* = 0.105, (**F**)). Data are presented as mean ± S.E.M. Sham animals were statistically different from all 6-OHDA-injured animals: # *p* ≤ 0.0001. Sham: n = 14–15 slices/5–6 animals; 6-OHDA: n = 16–20 slices/6 animals; NAC: n = 19–21 slices/6 animals. Abbreviations: dSTR: dorsal striatum; Ctr, contralateral; Ipsi, ipsilateral; NAC, N-Acetylcysteine; n.s: nonsignificant; SNpc: substantia nigra pars compacta; TH, tyrosine hydroxylase; 6-OHDA, 6-Hydroxydopamine. Magnification: 4× and 10×. Scale bar STR: 1000 μm. Scale bar SNpc: 10,000 μm.

Tyrosine hydroxylase staining works as an index of evaluation of the dopaminergic neuronal integrity after injury. Therefore, to address the impact of 6-OHDA injections and the resulting effects of the treatment, we proceeded to conduct a histological analysis using TH immunoreactivity (Figure 6A–F). Statistical analysis revealed the deleterious impact of 6-OHDA injections in DAn densities (F _(2, 47)_ = 65.151, *p* < 0.0001, η^2^_partial_ = 0.743) since a significantly decreased number of TH-positive cells was observed in the SNpc of 6-OHDA-lesioned animals when compared to the vehicle-treated animals (Sham, *p* <0.0001, Figure 6B,C). Overall, TH-positive densities in the nigra were not affected by the sole administration of NAC when compared to non-treated animals (*p* = 0.122, Figure 6B,C). Nevertheless, the counting of TH^+^ neurons in the SNpc exposed a general trend towards a higher distribution of TH^+^ cells (24.16%) in the ipsilateral lesioned side of NAC-treated animals than in the 6-OHDA group (9.70%; Figure 6D). The same tendency of results was observed in the TH-positive fiber labeling evaluation of the striatum. Statistical analysis revealed a significant impact of the factor treatment on the experimental groups (F _(2, 54)_ = 316.94, *p* < 0.0001, η^2^_partial_ = 0.921). Firstly, post hoc analyses demonstrated that all the animals injected with 6-OHDA displayed a remarkable decrease in TH-positive striatal fibers (Figure 6E,F) when compared to the control group (Sham, *p* < 0.0001, Figure 6E,F). However, as observed in the SNpc, the results disclosed that the administration of NAC did not enhance the preservation of TH-positive striatal fibers compared to the 6-OHDA group (*p* = 0.105, Figure 6E,F).

### 3.5. N-Acetylcysteine Administration Restored Dopamine Transporter (DAT) Levels upon 6-OHDA Lesion

**Figure 7:**
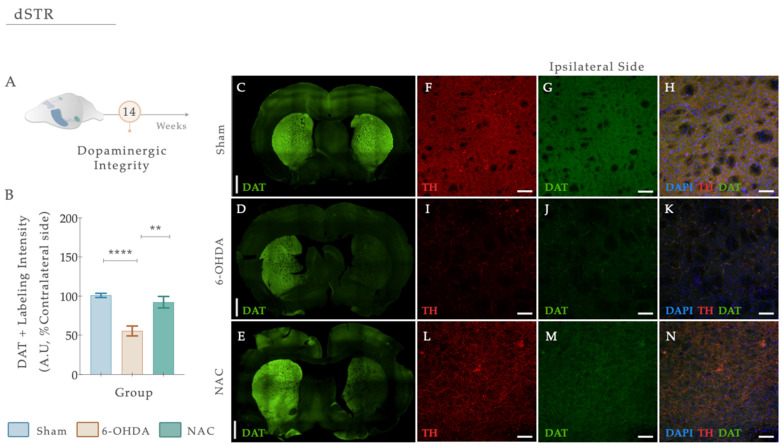
(**A**) N-Acetylcysteine administration re-established dopaminergic integrity. (**B**) Representative photomicrographs of immunofluorescence staining for the dopamine transporter (DAT) (green) in the dorsal striatum are observed (**C**–**E**). Confocal images of Tyrosine hydroxylase (TH) (red) and DAT (green) highlighting the dorsal striatum of the ipsilateral hemisphere and labeling the axonal terminals of dopaminergic neurons are shown in (**F**–**N**). A significant reduction in DAT levels was clearly visible in 6-OHDA-injected animals compared to the Sham group (**B**,**I**–**K**). After treatment application, NAC could modulate DAT levels compared to the 6-OHDA untreated group (**B**,**L**–**M**)**.** Data presented as mean ± S.E.M. **** *p* ≤ 0.0001. ** *p* ≤ 0.01. Sham: n = 4; 6-OHDA: n = 4; NAC: n = 4. DAT, dopamine transporter; Ipsi, ipsilateral; NAC, N-Acetylcysteine; dSTR, dorsal striatum; TH, tyrosine hydroxylase; 6-OHDA, 6-Hydroxydopamine. Magnification: 4× and 20×. Scale bars: (**C**–**E**): 1000μm; (**F**–**K**): 50μm.

DAT loss is highly evident at the level of the DAn axonal terminals after PD, functioning as a specific marker of DAn. Herein, we performed a double fluorescent staining for anti-TH (red) and anti-DAT (green) in striatal coronal brain sections (Figure 7A–E). From statistical analysis, we found that the injection of 6-OHDA alters dopaminergic integrity (Figure 7B; F_welch_ (2,48.02) = 21.869, *p* < 0.0001, η^2^_partial_ = 0.294). In fact, 6-OHDA animals revealed a significant reduction in DAT fluorescent intensity levels compared to the control group (Figure 7D,I–K; Sham; *p* < 0.0001; Figure 7). After applying our therapeutic pipeline, post hoc analysis revealed that NAC monotherapy could significantly increase the levels of DAT compared to the 6-OHDA untreated group (*p* = 0.0010; Figure 7E,L–N).

## 4. Discussion

Re-establishing DA levels, nigrostriatal function, and dopaminergic integrity (and, within it, regaining the sensory-motor functionality) is an unmet clinical requirement for developing new therapeutical strategies for PD [47]. In recent years, promising alternative strategies, such as disease-modifying/repurposing drugs, have been considered for PD. Under this concept of modality is N-acetylcysteine (NAC). This already-used pharmaceutical compound has demonstrated promising therapeutic effects in CNS applications and has been linked to some neuroprotective properties in the context of PD [27]. Nevertheless, even with these hypothetical and new concepts, a rational disease-modifying strategy still needs to be added to the PD therapeutic pipeline. Bearing this in mind, we believe that NAC application could be a remarkable asset by which to approach the disease, either alone or as an adjuvant to existing treatment options. As such, within the scope of the present work, we addressed the impact of NAC monotherapy application in a 6-OHDA intrastriatal animal model of PD.

To achieve such a goal, our initial experiments aimed to establish a striatal PD animal model induced by the unilateral injection of 6-OHDA in four different coordinates along the rostrocaudal axis of the ventrolateral striatum [39]. Such injection generates an intracellular oxidative stress environment, inducing DAn (retrograde) degeneration [48,49]. These lesions were unilaterally performed, reducing animal loss and producing motor impairments easily measured after the administration of dopaminergic agents, using the non-injected side as an internal control. As described, the main characteristic of this kind of animal model is that it resembles the oxidative nature of the DAn degeneration process as it occurs in human PD, leading to the appearance of the main motor symptoms of the disease [50]. Indeed, through the rotameter test, we verified that the 6-OHDA-injected animals presented a compromised dopaminergic system, since an apomorphine-induced turning behavior was observed compared to the Sham group (Figure 3). As the literature describes, the number of apomorphine-induced rotations reflects the extent of striatal DA depletion, in which higher rotation levels correlate with higher DAn loss/denervation [51]. Considering this, we were not expecting to see this significant rotation level in the lesioned animals, since Kirik et al. and other authors have shown that in striatal lesions, the stereotypic behavior is not observed or displayed a bimodal distribution [39,52,53]. However, in our results, the rotameter showed a moderate impairment, as the number of rotations reached an average of 52 turns. Compared with one of the conventional models using 6-OHDA, the medial forebrain bundle (MFB), these results are not so aggressive in promoting the degenerative process of DAn [45,54]. Such a compromising effect was also observed in animals’ motor coordination performance, as reaching abilities and forelimb/hindlimb use was affected (Figure 3). Indeed, the loss of reaching accuracy confirms and expands previous observations, where multiple authors have reported that skilled limb use impairments are correlated with nigrostriatal dopaminergic deficits [53,55,56,57]. Nevertheless, there have been, on the other hand, studies indicating that impairments in this type of behavior were not observed in striatal lesioned animals [39]. To justify this, Kirk et al. proposed that the profile of the behavioral deficits observed within this model variates according to the extent of the lesion, confirming that significant impairments can only be obtained with a four-coordinate model [39], such as the one used in this work. As far as we know, detailed reports on the staircase test have mainly focused on unilateral 6-OHDA nigrostriatal bundle lesions (MFB models). MFB lesion is one of the most common PD models; however, the use of this model can be criticized when employing neuroprotective strategies since few dopaminergic neurons reside in the nigrostriatal pathway upon lesioning [58]. Thus, the present outcomes enlarge the previous literature by thoroughly recounting skilled limb movements after striatal 6-OHDA injection. Moreover, in the manual foot misplacement corridor test, the obtained effects support the previous findings, as significant unilateral alterations of stepping patterns were observed (Figure 3). Using the irregular rung condition, our results identified consistent discrimination between the Sham and 6-OHDA groups, demonstrating that limb movements were impaired within stepping accuracy and posture. Most errors resulted from incorrect positioning of the lesion-impaired forelimb on the rung. Furthermore, Sham animals presented equal foot misplacements on both sides of the body, indicating perfect coordination of fore- and hindlimbs. In the event of a misstep, an unlesioned animal could preserve weight support, allowing foot placement errors to be quickly corrected and the foot to be replaced on the rung. Additionally, we also understood that the rat walking speed influenced locomotor parameters. Nevertheless, this measure did not accurately reflect the actual impairments. Although future considerations regarding this test should be appraised, according to the literature, this test represents a simple and reliable method for the assessment of the lesion severity in 6-OHDA animals and is in accordance with earlier studies describing substantial and long-lasting deficits in skilled limb use after unilateral striatal and cortical lesions [59,60]. Altogether, this part of the study disclosed stable behavioral readouts, mimicking early stages of motor symptoms in PD, thus indicating that this model could be used for studies aimed at neuroprotection, regeneration, or functional recovery in the nigrostriatal DA system. However, in prospective experiments, the analysis of the non-motor symptomatology of PD (depression, anxiety) through the sucrose preference test (SPT), the elevated plus maze (EPM), and the forced swim test (FST) should also be considered, as most of these dimensions typically precede the appearance of PD motor symptomatology, as previously described in similar models [57,61]. Nevertheless, even being a sustainable approach, to really reproduce the realistic concept of PD, the injection of αSyn particles/fibrils should be recommended since, in addition to leading to DAn degeneration, it also recreates one of the hallmarks of the disease, the accumulation of αSyn aggregates, and the consequent formation of Lewy bodies (LBs) [62]. With the successful establishment of the partial 6-OHDA intrastriatal PD model, our next purpose was to dissect the impact of NAC intervention, focusing on motor recovery, DAn cell survival, and DAT immunofluorescence profiles of 6-OHDA-injected animals. From the obtained results, we observed that after single-dose application, NAC led to a significant improvement in the skilled motor function of the animals, raising their ability to reach, retrieve, and successfully eat the pellets when compared to 6-OHDA-lesioned animals (Figure 4). The same proficient effect in reverting PD motor deficits was also observed in the forced-choice task (in the affected side), when compared to the 6-OHDA untreated group (Figure 4). Conversely, similar outcomes were perceived in the manual foot misplacement test, although therapeutical effects were only observed for left forelimb placement accuracy (Figure 5). At the histological level, our data demonstrated that reciprocal interactions between the applied therapy and 6-OHDA-lesioned brains only produced neurorescue effects on the striatum, with NAC providing significant protection against axonal degeneration. In fact, we saw that the administration of NAC did not increase the TH-positive neurons and fibers (Figure 6) but increased DAT fluorescence intensity (Figure 7), possibly indicating that our strategy could modulate dopaminergic transmission. Such results most likely mediated the positive motor functional gains that were noticed. Overall, our results align with the scientific and clinical literature, demonstrating that NAC could ameliorate and prevent performance failure in animal models of PD [63,64,65]. Importantly, in the clinics, studies have already demonstrated that the use of this drug (as a supplement) is correlated with noteworthy improvements in the Unified Parkinson’s Disease Rating Scale (UPDRS), denoting its positive impact on the amelioration of PD motor symptomatology [34]. Despite these interesting outcomes, the exact mechanism still needs to be elucidated. The modulation of GSH levels is described as one such hypothesis. Indeed, reports have suggested NAC as a booster of GSH synthesis in PD, increasing its levels in both animals’ and patients’ brains [32,66,67]. Nevertheless, it remains to be established whether GSH depletion is a primary cause of PD or a result of multiple deficiencies. Although not addressed in the present work, GSH is one of the most abundant soluble antioxidant agents present in the brain, indispensable for maintaining cell equilibrium as well as neuronal homeostasis [68]. Its synthesis requires three types of amino acids: glutamate, supplied by astrocytes, cysteine (Cys), and glycine, which are also derived from astrocytes [69]. GSH is typically synthesized in the cytoplasm of cells and particularly relies on the influx of Cys in order to carry out the rate-limiting step of GSH synthesis [70]. In neurons, Cys is transported via the excitatory amino acid transporter C1 (ECAAC1) [71]. However, to supplement the intracellular thiol pool, this tripeptide also employs cystine/glutamate (Xc−) antiporters, commonly present in astrocytes [72]. Actually, it has been acknowledged that the higher production of GSH occurs in astrocytes, rather than in neurons, making these glial cells essential during this process [73]. According to a review by Deponte, several detoxifying cellular enzymes use GSH as a substrate or cofactor, comprising glutathione reductase, glutaredoxin (Grx), glutathione peroxidase, peroxiredoxin (Prx), glyoxalases 1 and 2, glutathione transferase, and MAPEG (membrane-associated proteins in eicosanoid and glutathione metabolism) [74]. In addition to this, GSH is also broadly involved in the cellular removal of H_2_O_2_ and other hydroperoxides [74]. Although GSH is not the only molecule reported to be altered in PD, the magnitude of its depletion is correlated with the severity of the disease [75]. In the substantia nigra of patients, within the surviving DAn, there is a remarkable decline of GSH, thus having the status of the earliest known biochemical indicator of nigral degeneration [76]. In accordance, further studies have implied that various enzymes composing the GSH system appear to be modified in the PD brain, including glutathione peroxidase and glutathione-S-transferase [77]. Importantly, the depletion of GSH has also been attributed to the occurrence of mitochondrial dysfunction, particularly the decline of mitochondrial complex I activity in brain cells [78]. Actually, when an oxidative condition, such as the one observed in our 6-OHDA model, affects the cellular content of GSH and, consequently, the natural defense, then a rational therapeutic intervention could be based on replenishing GSH. By enhancing the intracellular cysteine pool (central to brain GSH synthesis), NAC can enhance GSH levels in the brain of PD animals [63,66] and patients [32]. Once inside the cells, N-acetyl-l-amino acids are deacetylated by several aminoacylases (I, II, and III), and NAC is, thus, hydrolyzed by cytosolic acylase I [79]. In normal conditions, the concentration of NAC required to facilitate equivalent oxygen-centered radical scavenging is larger than that of GSH [80], which possibly indicates that NAC might not have direct antioxidant mechanisms. Indeed, as described elsewhere, the ability of NAC to scavenge one of the major biologically relevant radical species, superoxide, is not detectable [81]. In this context, it is extremely likely that NAC’s antioxidant effects are mediated by increased levels of intracellular GSH. This distinction is relevant since it denotes that certain conditions must be met before NAC can confer antioxidant properties. In fact, an intact enzyme machinery (glutamylcysteine synthetase; glutathione synthetase) and a functional cystine uptake system (cystine/glutamate antiporter) are required [82]. Considering these findings, one could reasonably conjecture that there might be additional mechanisms, indirectly connected to NAC’s actions, that solely become activated upon GSH production and stimulation. Since GSH can bind to DA quinone (a DA neuron-specific oxidative stress agent) via its thiol group and suppresses DA quinone toxicity, higher levels of GSH might not only quench general ROS, but also DA quinones [83]. Adding to this, the glutathione-related redox state obtained after NAC administration could also play an important role in the regulation of cytokine signaling, including tumor necrosis factor-α (TNFα), interleukin (IL)-1β and IL-6, and the modulation of immune-inflammatory pathways [84,85]. Moreover, other cellular processes could also be synchronized with the anticipated elevation of GSH. For instance, as neuronal GSH synthesis is largely dependent on astrocytes, it is plausible that NAC, through its ability to upregulate GSH supply or by interacting with DAT, may also have an impact on these glial cells. Therefore, it will be essential to account for the analysis of these specific cells in the future, as modifications in their neuroprotective mechanisms could strengthen our findings.

In addition, we observed that NAC sole administration increased dopaminergic transmission through the increase in DAT fluorescence intensity. Indeed, such an effect had already been reported by Monti et al., who found an increase in DAT binding expression (through DaTscan) in the caudate and putamen of PD patients after NAC administration [34]. In support of this, Hashimoto et al. showed that NAC could delay the reduction of DAT in the striatum of monkeys after repeated methamphetamine administration (linked with neurotoxicity of dopaminergic nerve terminals similar to what occurs in PD) [86]. Based on this, it is proposed that this drug may positively affect the dopaminergic system.

Despite these promising and potential observations, some discrepancies should be considered. Regarding the manual foot task, the absence of foot-faults score differences observed in the left side of both fore- and hindlimbs (Figure 5) could be justified by the scoring system applied. This method cannot track movements, with the high resolution being largely qualitative [87]. Among hindlimbs, results clearly reflect an increase in basic locomotor function during walking (Figure 5), which could likely be related to anatomical differences in limbs or to the fact that the hindlimb may not be essential for locomotor control under these conditions [88]. While impaired motor phenotype in the 6-OHDA animals was intensified during the behavioral assessment, NAC only stabilized misstep quantification after 4 weeks of treatment on forelimb placement accuracy (Figure 5), thereby suggesting that this drug could have a notable effect on the underlying processes of the disease’s continued neurodegeneration in a later period, rather than at an early stage following administration. Remarkably, this potential is consistent with the effects obtained in the staircase, where NAC only appears to have a significant impact after 4 weeks following administration (Figure 4). Suboptimal dosages and routes of administration may account for some of these results. After oral administration, NAC is rapidly absorbed, presenting a terminal half-life of 6.25 h and a total oral bioavailability that varies between 6–10% [89]. As previously mentioned, NAC was acutely given by oral gavage, indicating that to reach the brain, it must pass through the systemic circulation and posteriorly cross the BBB. When reaching the PD-damaged brain regions, as NAC enters the neurons, it is metabolized to release cysteine, a rate-limiting component for the de novo synthesis of GSH. Second, messengers and modulators can be activated, regulating intrinsic signaling pathways and cellular survival processes [90]. As such, this process, which endures for some time, might promote a late effect on the BG-affected areas and mechanisms, thus gradually modulating movement control. In line with this are the results obtained with DAT, which indicate that 5 weeks after treatment, it is still possible to see a modulating effect of the drug (Figure 7). The induced reestablishment of GSH levels could, in turn, indirectly increase extracellular DA levels in the synaptic cleft [91]. Another potential mechanism through which NAC could modulate the transport and metabolism of DA that should not be ignored and has only recently been considered—is cysteine proteome regulation [92,93]. In the cytoplasmic membrane of the DAT, there are eight cysteine residues, with some being vital for ensuring the three-dimensional structure and function of the transporter [94,95]. With this in mind, NAC may modulate dopamine transport and metabolism via direct interaction with sensitive cysteine residues within the transporter, since it has disulfide-breaking activity [96,97]. Furthermore, another unexpected result was the one observed in TH-positive labeling, with NAC displaying an absence of significance both in SNpc and striatum, respectively. As TH is subject to stress-induced changes [98], downregulation could result from decreased sensitivity to the binding method. If TH levels increase and decrease in individual neurons through stress-induced changes in gene expression, measurements of overall TH expression may not be in proportion to changes in the nigrostriatal area [99]. In addition, TH is not a complete marker of DAn, masking the remaining DAn, and thus, hiding the NAC pharmacologic effect. As a result, TH^+^ immunolabeling may not always reflect a true loss or gain of dopaminergic terminals/somas. This could also justify the results obtained in TH^+^ counts, indicating that the number of cells increases with the treatment, despite not being measurable (Figure 6D). These observations were quite puzzling, as reports demonstrate that, in contrast to the striatum, NAC does not promote significant protection of the somata, as overall TH expression in the nigra is not modified [100]. Still, as previously assumed, some researchers have supported the protective influence of NAC against induced parkinsonism [38,101,102,103], revealing that, several days after a severe oxidative insult, NAC can provide long-term neuroprotection of striatal TH fibers [100,104,105]. Moreover, research from Virel and colleagues also indicated that at an early time point, NAC does not protect striatal dopaminergic fibers against 6-OHDA insult [106], which pinpoints that regeneration of the lesioned striatum may be achieved at later stages, as reported for other antioxidants [107]. Hence, we believe that multiple time points and different treatment pipelines are necessary to really address NAC’s temporal and therapeutic effects. In parallel with this, substantial data from psychiatric and addiction studies have shown that NAC can act on the CNS by directly and indirectly modulating glutamatergic neurotransmission and its homeostasis [108]. In the ventral striatum, DA neurons make the most robust glutamate (Glu) connections [109]. Accordingly, vesicular synergy (one neurotransmitter potentiates the uptake of another neurotransmitter) is thought to occur between these two types of neurons, enabling DAn to co-transmit glutamate (DA-Glu neurons) [110]. As a result, reports have shown that: (1) 6-OHDA injections in the dorsal striatum increased the number of DA-Glu neurons [111], and (2) 1-methyl-4-phenyl pyridinium (MPP^+^) exposure in mouse DA neuron culture increased the VGluT2 copy number per cell, whereas the TH copy number per cell was reduced [112]. Thus, DA and glutamate appear to have a reciprocal functional interaction in the striatal motor complex. This indicates that agents capable of modulating glutamatergic transmission may represent an approach to managing conditions associated with dopaminergic excitotoxic neuronal dysfunction. As such, we can speculate that NAC supplementation might act particularly in these neurons in preclinical models of PD, just like this one, either by the alteration of extra-synaptic mGlu2/3 receptors (located on neuronal terminals) [113] or by inhibiting NMDA-mediated increases in intracellular calcium levels via GSH [114]. In fact, the modulatory action of NAC on glutamate homeostasis is perhaps the best characterized [115]. By activating cysteine/glutamate ateantiporter (Sxc–; a key component in the control of extracellular glutamate) and consequently activating mGlu, NAC has been shown to counteract excessive and neurotoxic glutamate release from excitatory nerve endings [116]. As such, we believe that the effects of NAC presented here could be associated with glutamate modulation in the striatum, already reported in a 6-OHDA model [117], with an indirect impact on DA transporter levels. Hence, future analysis of VGLUT-2, mGluRs, and Sxc– should be considered and correlated with histological and behavioral data.

Altogether, upcoming studies should be performed addressing the temporal effects of NAC. Notably, alterations in the treatment regimen, including the route and frequency of administration, the volume of injection, and the dose application timeline, should be considered. Along the same line, unveiling in vitro target levels and target occupancy (saturation) of the NAC, in addition to a monitorization of the plasma/brain concentration ratio, could be relevant for the study, giving insights into their potential mechanistic effects. Models such as the BBB-on-chip and inducible pluripotent stem cells (iPSCs)-derived DAn [118] would be very helpful for drug screening studies using NAC, to provide high-resolution temporal readouts of barrier permeability and barrier-protective effects [118]. Since limited information is available regarding NAC’s effects on neuroinflammation, investigating the dynamic interplay between inflammatory cells under this therapeutic condition might be important. Furthermore, DA receptor regulation should be explored, as emerging research has suggested that a complex interplay between DAT and DA receptors occurs, along with changes in receptor expression and signaling pathways, which often challenges PD pathophysiology and treatment [119]. In fact, in the absence of pathology, the evidence disclosed a direct protein–protein interaction between the D2 receptor and DAT [120]. This D2–DAT coupling may constitute an important event, facilitating DAT upregulation, and resulting in the spatial and temporal regulation of DA signaling and, consequently, normal dopaminergic neurotransmission [119,121]. Still, the assessment of other molecular changes such as SOD-1, iNOS, catalase [102,122], as well as total JNK, NF-kB [38], and inflammatory cytokine levels [123] should also be measured, given their involvement in NAC mechanisms and signaling pathways. By doing so, new avenues with important gains in PD therapeutics may be opened up, with a potential translation into clinics.

## 5. Conclusions

Currently, PD presents a multifactorial profile with many actors simultaneously operating to compose a very complex scenario, manifesting itself through various pathophysiological processes. Alongside this, the accessible therapeutic approaches for PD, operating through the restoration of dopaminergic tone, mainly offer symptomatic palliative relief, and after a few years of treatment, motor complications and dyskinesias inevitably emerge. In addition, none of the existing treatment options can prevent or halt the processes underlying the disease’s progressive state or treat the non-dopamine-dependent features of PD. In retrospect, with the analysis performed in this study, we provided an important proof-of-concept milestone regarding the therapeutical application of NAC, since we detected promising outcomes in the modulation of PD motor and histological deficits. Nonetheless, although NAC has already been shown to be beneficial in multiple neurological conditions, it continues to be important to carefully characterize and define its targets. Therefore, it is imperative to understand the complex capacity of NAC and how its therapeutic neuroprotective effects interact with cellular and molecular PD mechanisms. Indeed, following such a research approach will allow for the exploration of not only the potential circuitries involved in the disease’s recovery and/or compensation mechanisms, but also develop a targeted-based strategy that can generate potential clinical benefits, and hence, be translated for PD patients.

## Data Availability

Data is contained within the article or Supplementary Material.

## Supplementary Materials

The following supporting information can be downloaded at: https://www.mdpi.com/article/10.3390/antiox12061257/s1, Figure S1: Impact of NAC administration under paw reaching right forced performance task, 1 and 4 weeks upon treatment administration; Figure S2: Beneficial effects of NAC intervention on right locomotor function, 1 and 4 weeks upon treatment administration.
