# Supplementary material for: N-Acetylcysteine Treatment May Compensate Motor Impairments through Dopaminergic Transmission Modulation in a Striatal 6-Hydroxydopamine Parkinson’s Disease Rat Model
Source: Antioxidants (Basel). 2023 Jun 11;12(6):1257. doi: 10.3390/antiox12061257 (PMC10295771; doi:10.3390/antiox12061257)
Supplement: Supplementary file 1 [file antioxidants-12-01257-s001.zip › antioxidants-2358614-supplementary.pdf]

Staircase - Forced Choice Right

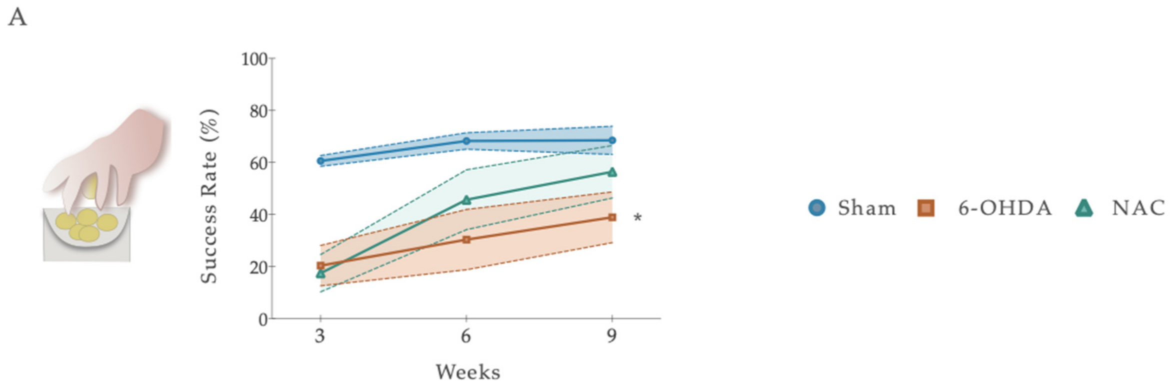

Figure S1. Impact of NAC administration under paw reaching right forced performance task, 1 and 4 weeks upon treatment administration. A significant impairment was observed when comparing the 6-OHDA animals with the control group (p= 0.028). However, no differences were found with NAC administration (p= 0.293) (Mixed design factorial: Treatment: $F_{(2, 22)} = 7.694$, $p = 0.003$; $\eta^2_{partial} = 0.412$; Time: $F_{(2, 44)} = 13.51$, $p < 0.0001$, $\eta^2_{partial} = 0.380$; Interaction: $F_{(4, 44)} = 2.436$, $p = 0.061$, $\eta^2_{partial} = 0.061$. Data presented as mean ± S.E.M. For the tests: Sham: n = 9; 6-OHDA: n = 8; NAC: n = 8. NAC, N-Acetylcysteine; 6-OHDA, 6-Hydroxydopamine.

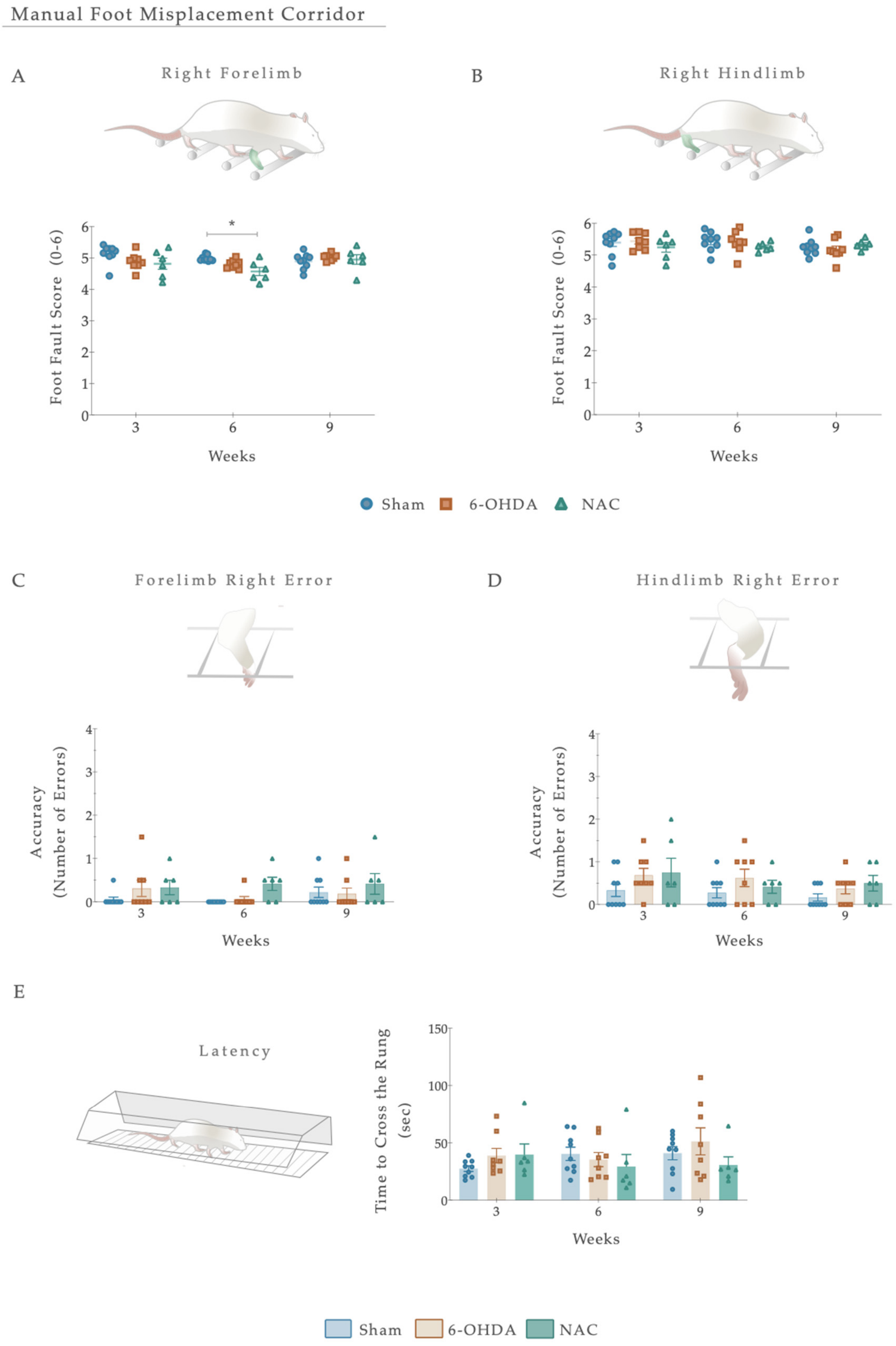

Figure S2. Beneficial effects of NAC intervention on right locomotor function, 1 and 4 weeks upon treatment administration. (A) Results revealed that forelimbs on the ipsilateral (right) side demonstrated minimal changes in

the foot fault scores. Two-way ANOVA: Treatment: $F_{(2,20)} = 4.731$, $p = 0.021$; $\eta^2_{partial} = 0.321$; Time: $F_{(2, 40)} = 2.794$, $p=0.073$, $\eta^2_{partial} = 0.123$; Interaction: $F_{(4, 40)} = 2.061$, $p = 0.104$, $\eta^2_{partial} = 0.171$). (B) Similar results were observed when comparing the right hindlimbs of all animals through the different time-points (Two-way ANOVA: Treatment: $F_{(2, 20)} = 0.275$, $p = 0.763$; $\eta^2_{partial} = 0.027$; Time: $F_{(2, 40)} = 1.366$, $p = 0.267$, $\eta^2_{partial} = 0.064$; Interaction: $F_{(4, 40)} = 0.981$, $p = 0.429$, $\eta^2_{partial} = 0.089$). (C) Accuracy of placement on the ipsilateral forelimbs did not differ between the groups when analyzed (Two-way ANOVA: Treatment: $F_{(2, 20)} = 6.230$, $p = 0.008$; $\eta^2_{partial} = 0.384$; Time: $F_{(2, 40)} = 0.500$, $p = 0.611$, $\eta^2_{partial} = 0.024$; Interaction: $F_{(2, 40)} = 0.513$, $p = 0.726$, $\eta^2_{partial} = 0.049$). (D) Adding to this, the number of errors on the right hindlimbs were similar in all groups (Two-way ANOVA: Treatment: $F_{(2, 20)} = 1.180$, $p = 0.328$; $\eta^2_{partial} = 0.106$; Time: $F_{(2, 40)} = 1.164$, $p = 0.323$, $\eta^2_{partial} = 0.055$; Interaction: $F_{(4, 40)} = 0.986$, $p = 0.426$, $\eta^2_{partial} = 0.090$). (E) No significant effects of NAC treatment on latency to cross the apparatus was perceived ($p > 0.05$; Treatment: $F_{(2, 20)} = 0.719$, $p = 0.499$, $\eta^2_{partial} = 0.067$; Time: $F_{(2, 40)} = 0.742$, $p = 0.483$, $\eta^2_{partial} = 0.036$; Interaction: $F_{(4, 40)} = 1.367$, $p = 0.263$, $\eta^2_{partial} = 0.120$). Data presented as mean ± S.E.M. For the tests: Sham: n = 9; 6-OHDA: n = 8; NAC: n = 6. NAC, N-Acetylcysteine; sec, seconds; 6-OHDA, 6-Hydroxydopamine.
